# Measuring the lactate-to-creatine ratio via ^1^H NMR spectroscopy can be used to noninvasively evaluate apoptosis in glioma cells after X-ray irradiation

**DOI:** 10.1186/s11658-018-0092-2

**Published:** 2018-06-15

**Authors:** Hongxia Li, Yi Cui, Fuyan Li, Wenqi Shi, Wenjing Gao, Xiao Wang, Qingshi Zeng

**Affiliations:** 1 Department of Radiology, the Second Hospital of Shandong University, Jinan, China; 2 Department of Radiology, Qilu Hospital of Shandong University, 107 Wenhuaxi Road, Jinan, 250012 China; 3 Department of Radiology, Shandong Medical Imaging Research Institute, Jinan, China; 4 Department of Radiology, the Third Affiliated Hospital, Sun Yat- Sen University, Guangzhou, China

**Keywords:** DNA damage, P-PKC-ι, Lactate, Glioma, X-ray irradiation, Apoptosis

## Abstract

**Background:**

Radiotherapy is among the commonly applied treatment options for glioma, which is one of the most common types of primary brain tumor. To evaluate the effect of radiotherapy noninvasively, it is vital for oncologists to monitor the effects of X-ray irradiation on glioma cells. Preliminary research had showed that PKC-ι expression correlates with tumor cell apoptosis induced by X-ray irradiation. It is also believed that the lactate-to-creatine (Lac/Cr) ratio can be used as a biomarker to evaluate apoptosis in glioma cells after X-ray irradiation. In this study, we evaluated the relationships between the Lac/Cr ratio, apoptotic rate, and protein kinase C iota (PKC-ι) expression in glioma cells.

**Methods:**

Cells of the glioma cell lines C6 and U251 were randomly divided into 4 groups, with every group exposed to X-ray irradiation at 0, 1, 5, 10 and 15 Gy. Single cell gel electrophoresis (SCGE) was conducted to evaluate the DNA damage. Flow cytometry was performed to measure the cell cycle blockage and apoptotic rates. Western blot analysis was used to detect the phosphorylated PKC-ι (p-PKC-ι) level. ^1^H NMR spectroscopy was employed to determine the Lac/Cr ratio.

**Results:**

The DNA damage increased in a radiation dose-dependent manner (*p* < 0.05). With the increase in X-ray irradiation, the apoptotic rate also increased (C6, *p* < 0.01; U251, *p* < 0.05), and the p-PKC-ι level decreased (C6, *p* < 0.01; U251, *p* < 0.05). The p-PKC-ι level negatively correlated with apoptosis, whereas the Lac/Cr ratio positively correlated with the p-PKC-ι level.

**Conclusion:**

The Lac/Cr ratio decreases with an increase in X-ray irradiation and thus can be used as a biomarker to reflect the effects of X-ray irradiation in glioma cells.

## Background

Glioma is one of the most common types of primary tumors of the central nervous system, and high-grade gliomas, especially glioblastomas, have aggressive behavior, rapid growth and poor prognoses [1]. The majority of patients die within 1 or 2 years of diagnosis [2, 3].

Radiotherapy after surgery can reduce the risk of tumor recurrence and significantly prolong survival time, but the mechanism underlying this remains unclear. Previous studies demonstrated that DNA damage could induce tumor cell apoptosis, and that this is associated with protein kinase C (PKC) activation [3–7]*.* Furthermore, recent advances in the study of tumors show that PKC iota (PKC-ι) expression is highly upregulated in tumor cells [4, 8]. Notably, Yang et al. suggested that the in vitro radiosensitizing effects of tamoxifen on glioma cells were partly caused by the inhibition of PKC-ι activity [4]. We also recently demonstrated that X-ray irradiation could lead to inhibition of PKC-ι activity, and that this correlated with the radiosensitivity of the cells [9].

The DNA damage response, which is essential for the survival of tumors, is related to cell cycle blockage and apoptosis [10, 11]. In human glioma cell line U251, X-ray irradiation causes DNA damage, cell cycle blockage and apoptosis [12, 13]. Therefore, X-ray irradiation could increase DNA damage, causing G1 stage blockage and cell apoptosis. Cerne et al. demonstrated that ionizing radiation decreased the viability, proliferation and clonogenic potential of tumor cells [14].

The lactate-to-creatine (Lac/Cr) ratio can be used as a biomarker of various cancer cell processes, because the lactate level rises with the proliferation of tumor cells [15–17]. In this study, to establish whether the Lac/Cr ratio can be used as a noninvasive monitor for radiotherapy, we evaluated the correlation between this ratio, the PKC-ι expression level and apoptosis in glioma cells.

## Methods

### Cell culture

Glioma cell lines (C6 and U251) were maintained in Dulbecco′s modified Eagle′s medium and replenished with 10% fetal bovine serum (FBS). The cells were cultured at 37°C in an atmosphere containing 5% CO_2_. The medium was replaced at intervals of 2 days and the cell condition was observed every 8 h.

When cell density reached 70%, the cells were randomly separated into 5 groups and each group was subjected to a different dose of X-ray irradiation: 0 Gy for the control group and 1, 5, 10 or 15 Gy for the treatment groups. After exposure, each group was separated into 4 sub-groups: one to be evaluated using single cell gel electrophoresis (SCGE) to determine DNA damage (tail moment); one to undergo flow cytometry to detect the cell apoptotic rate and cycle distribution; one to undergo western blotting to detect the p-PKC-ι expression level; and one to be assessed with ^1^H NMR spectroscopy to determine the Lac/Cr ratio. The experiment was performed three separate times.

### SCGE

The DNA damage induced by X-ray irradiation was analyzed via SCGE using a comet assay kit (Cell Biolabs, Inc.) at high pH. The cells were mixed with molten agarose. The DNA was relaxed and denatured using a lysis buffer and an alkaline solution. After the electrophoresis of these samples, they were dried and stained, and then observed using epifluorescence microscopy. The damaged DNA was found to migrate further than the intact DNA and then form a comet-shaped structure.

The degree of DNA damage was evaluated by measuring the displacement between the genetic material of the “comet head” (the nucleus of the cell) and the resulting “comet tail” (the DNA that has escaped from the nucleus). For every sample, we assessed the DNA damage for 50 cells. The tail moment is a typical index that considers both the migration of the genetic material and the relative amount of DNA in the tail. The % of DNA in the tail is calculated thus:

$$ \%\mathrm{tail}\ \mathrm{DNA}\ \left(\mathrm{TDNA}\right)=100\times \mathrm{tail}\ \mathrm{DNA}\ \mathrm{intensity}/\mathrm{cell}\ \mathrm{DNA}\ \mathrm{intensity} $$

The tail moment is calculated thus, based on the formula supplied with the Cell Biolabs comet assay kit:

$$ \mathrm{Tail}\ \mathrm{moment}=\mathrm{TDNA}\times \mathrm{length}\ \mathrm{of}\ \mathrm{tail} $$

The data were analyzed using CometScore (TriTek Corp.).

### Flow cytometric analysis of cell cycle distribution

The effects of X-ray irradiation on C6 and U251 cell cycle distribution were assessed using flow cytometry. After irradiation, the cells were incubated for 24 h, digested and washed twice with ice-cold phosphate-buffered saline (PBS). The cell density was adjusted to 2 × 10^6^ cells/ml with PBS. The cells were washed 3 times with ice-cold PBS and then fixed with 70% ethanol overnight at 4°C and incubated with propidium iodide for 30 min at 25°C in the dark. The samples were analyzed using a Becton, Dickinson and Company flow cytometer, and the data were processed using FlowJo (version 7.6, Tree Star Inc.).

### Flow cytometric analysis of apoptosis

After irradiation, the cells were washed 2 times with ice-cold PBS and then diluted to 1 × 10^6^ cells/ml with a 1× binding buffer. About 100 μl of the solution (1 × 10^5^ cells) was poured into a culture tube and 5 μl of FITC Annexin V and 5 μl of propidium iodide were mixed in. The cells were gently shaken, cultured for 15 min at 25°C in the dark, and 1 × binding buffer at 400 μl was added to each sample. The samples were then analyzed using an FITC Annexin V Apoptosis Detection Kit I (BD Biosciences).

### Western blot analysis

The cells were resuspended in RIPA buffer consisting of 50 mMTris-HCl (pH 8.0), 150 mMNaCl, 1.0% NP-40, 0.5% sodium deoxycholate, 0.1% SDS and 2 mM EDTA, supplemented with protease and phosphatase inhibitors and dithiothreitol and then sonicated in an ultrasonic washer. The protein content of each sample was assessed, and the proteins were denatured. The buffer, consisting of Tris 0.5 mM (pH 6.8), 50% glycerol, 10% SDS, 10% 2β-mercaptoethanol and blue bromophenol, was added to each sample at a ratio of 1:1. The protein extracts were then heated at 98°C for 10 min before use. For the western blot analysis, 10 μg of protein was separated on 10% SDS-PAGE and then transferred to a polyvinyl difluoride (PVDF) membrane. The membrane was soaked in 5% nonfat milk solution for 1 h at room temperature and incubated overnight at 4°C with rabbit anti-PKC iota (orb312760; 1:1000; Biorbyt), rabbit anti-phosphorylated PKC iota (ab5813; 1:1500; Abcam) and mouse anti-beta actin (TA-09; 1:1000; ZSGB-BIO). The first antibody solution was removed from the membrane, which was then washed 3 times with TBST, and incubated for 1 h at room temperature with peroxidase-conjugated affinipure secondary antibodies (1:1000; ZSGB-BIO). An enhanced chemifluorescence system was used to observe the immunoreactivity. The protein expression was quantized using Image Quant TL for Windows ver. 2005 (Amersham Biosciences). The expression of β-actin was used as a loading control for PKC-ι and p-PKC-ι [18].

### Sample preparation for ^1^H NMR spectroscopy

We removed the medium from the cells after X-ray irradiation. We then scraped the cells from the 175-cm^2^ culture dishes and washed them with ice-cold PBS. The cells were centrifuged for 5 min at 1000 rpm. The medium was discarded, and the cells were washed 2 times with ice-cold PBS.

The cells from two 175-cm^2^ culture dishes (approximately 3 × 10^7^ cells) were mixed inperchloric acidand triturated. Sodium hydroxide was used to adjust the pH of the samples to 7.2, and the samples were centrifuged using a Beckman Coulter Optima L-100 XP for 25 min at 18,000 g and 4°C. The precipitate was discarded and the supernatant was lyophilized. The lyophilized extracts were a powder that was put into 5-mm MR tubesand dissolved in pure 540 μl D_2_O. About 60 μl D_2_O containing 0.1% 2, 2, 3, 3-d (4)-3-(trimethylsilyl) propionic acid sodium salt (TMSP) was added before the test. D_2_O was used for the field frequency lock and TMSP was used to provide the chemical shift reference (delta chemical shift = 0.00 ppm). All procedures were conducted at 4°C. Three replicates were prepared for each condition [19].

### ^1^H NMR spectroscopy

All ^1^H NMR spectroscopy experiments were conducted on a BrukerAvance 600 MHz spectrometer. Solvent-suppressed 1D NOESY spectra were acquired. Tuning and matching were conducted for each sample. Shimming was completed automatically. A pulse–acquire sequence was used to collect 128 scans. The TMSP peak (0.00 ppm) was used as a reference signal. We determined the metabolite concentrations relative to the sum of all quantified metabolites and obtained the metabolite quantities using the MestReNova software (MestReC of Santiago de Compostela University) [20]*.* The Cr content changed slightly in the glioma cells, and we used the Lac/Cr ratio to determinethe relative lactate content.

### Statistical analysis

The data were analyzed using SPSS17.0 (SPSS Inc.). All data were presented as mean values±standard deviation. The differences between the DNA damage, apoptotic rates, p-PKC-ι expression and Lac/Cr ratios of the 2 cell lines were assessed using an independent-samples t test or ANOVA. We then used the results to plot a graph with SigmaPlot 12.0 or GraphPad Prism5.0. The significance level was set to 0.05.

## Results

### DNA damage

**Fig. 1:**
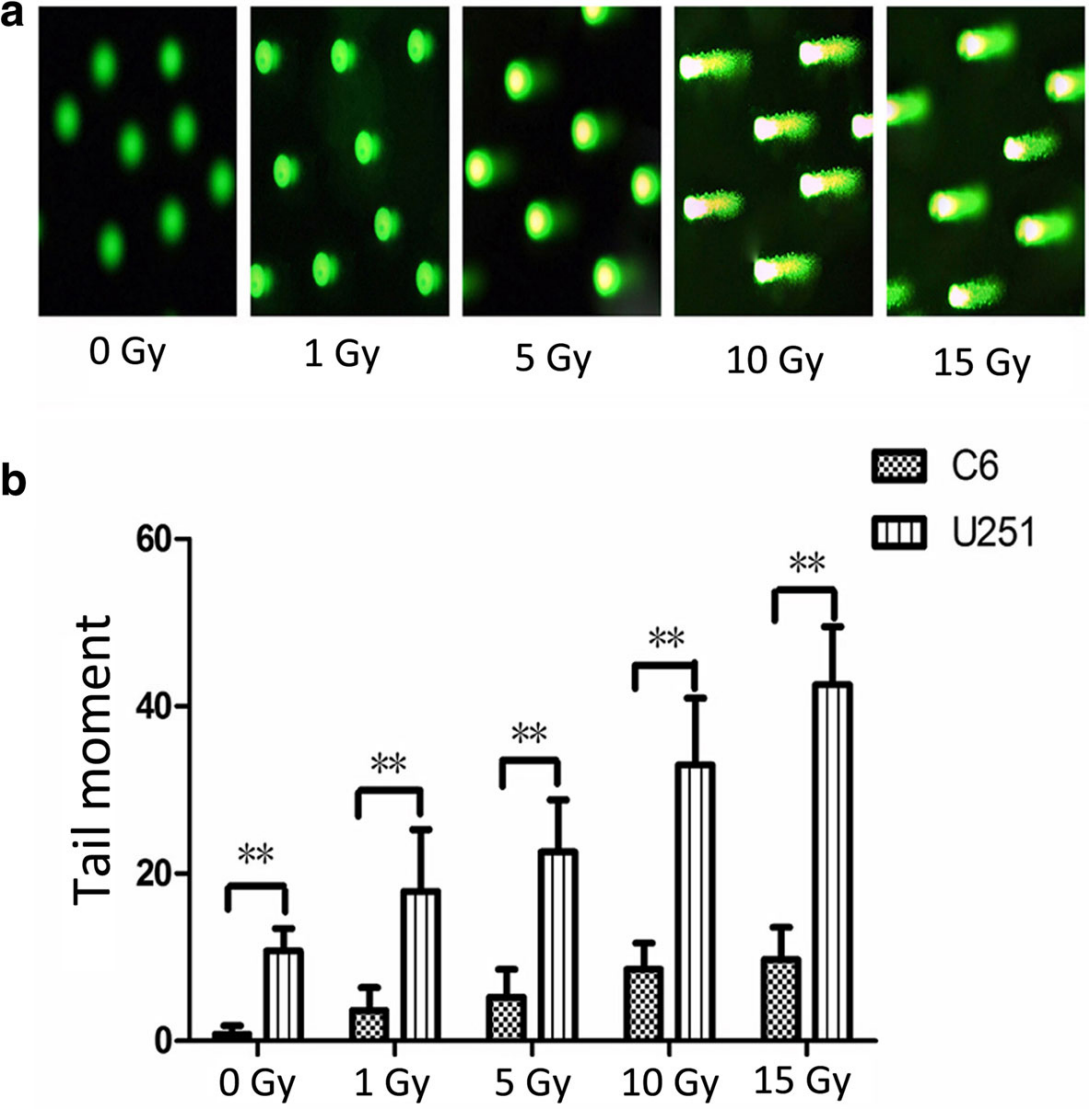
Results of the single cell gel electrophoresis (SCGE). **a** – Representative images of C6 cells after SCGE. The “comet tail” became longer as the dose of X-ray irradiation increased, indicating that DNA damage induced by X-ray irradiation at 1, 5, 10 and 15 Gy was more severe than that at 0 Gy (*p* < 0.05). **b** – The tail moments of U251 cells were larger than those of C6 cells when the cells were exposed to 0, 1, 5, 10 and 15 Gy X-ray irradiation. **p < 0.01

We evaluated the DNA damage using SCGE. Representative images of C6 are found in Fig. 1a. The “comet tail” became longer as X-ray irradiation increased, indicating that the DNA strand breaks were induced by X-ray irradiation. The tail moment increased in a radiation dose-dependent manner, as shown in Fig. 1b. The tail moments of U251 were larger than those of C6 at all levels of exposure. Significant differences were indicated (*p* < 0.01).

### Cell cycle distribution

**Fig. 2:**
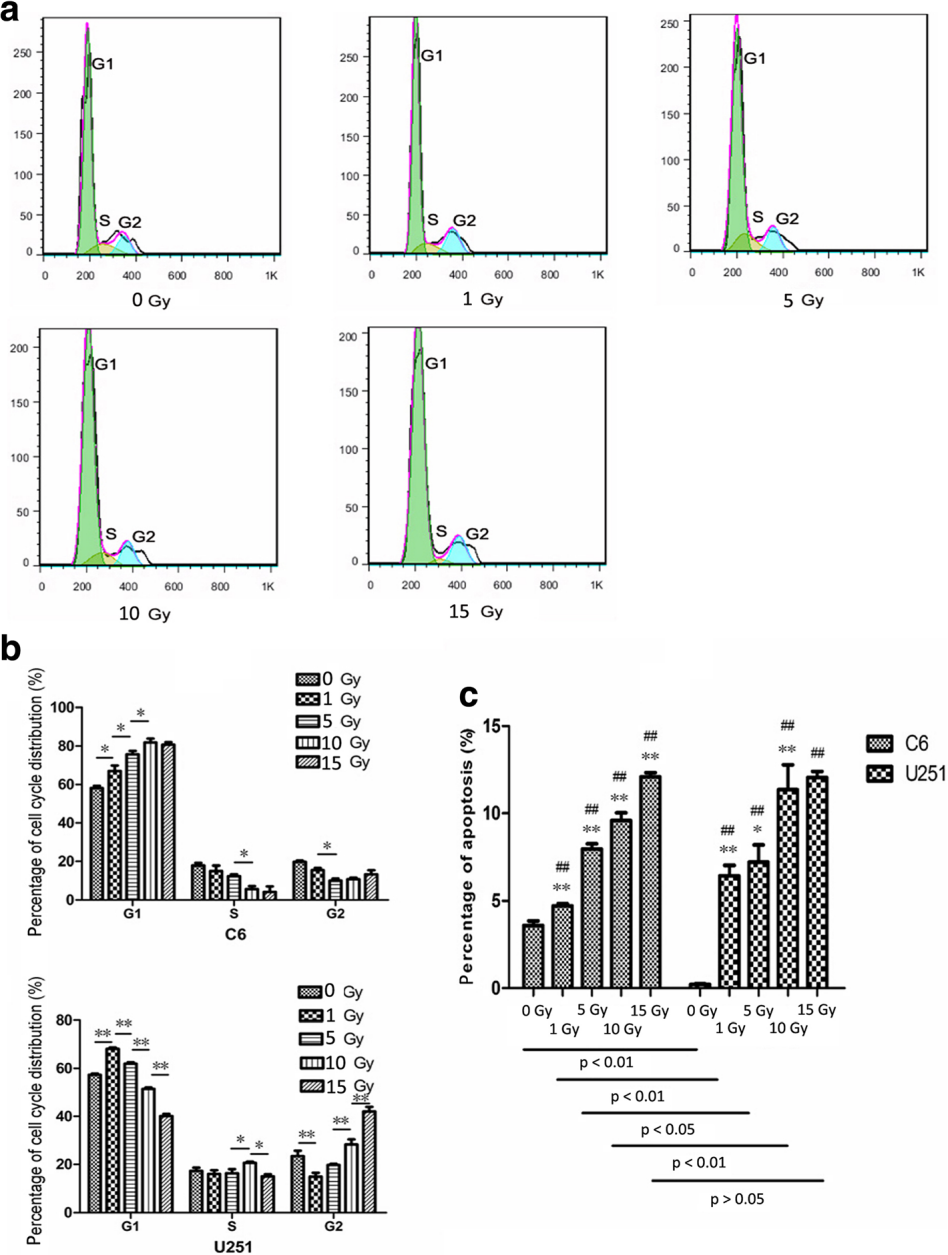
Cell cycle distribution and apoptotic rates of the two cell lines. **a** – Flow cytometry was performed and data were analyzed using FlowJo 7.6. A representative series of cell cycle FACS histogramsfor C6 cells are shown. The cell cycle distribution (**b**) and apoptotic rate (**c**) were assessed. **b** – The percentage of cells at each stage of the cell cycle are shown for C6 and U251 cells.*p < 0.05, **p < 0.01. **c** – The apoptotic rate increased with increasing X-ray irradiation. *p < 0.05, **p < 0.01: comparisons with the previous group (e.g., 5 Gy and 1 Gy; 10 Gy and 5 Gy); ^#^*p* < 0.05, ^##^*p* < 0.01: comparisons with the control group. The apoptotic rates of C6 were also compared with those for U251 cells for the same radiation dose. Statistical significance was determined as *p* < 0.05. This is shown in the bars under the x-axis

Flow cytometry was employed to investigate the cell cycle distribution of glioma cells. A series of representative FACS histogramsof C6 arepresented (Fig. 2a). For the C6 cells exposed to doses between 0 and 10 Gy, the percentages of cells inG1 increased with increasing X-ray irradiation (Fig. 2b).The values were 58.12%±1.98% (0 Gy), 67.08%±4.79%(1 Gy), 75.72%±2.97%(5 Gy), and 81.86%±3.43%(10 Gy). For all results, *p* < 0.05. When the cells were exposed to 15 Gy, the percentage in G1 phase was 80.72%±2.35%, which was not significantly different from the value for 10 Gy (*p* > 0.05).

For the U251 cells, the percentages of cells in G1 phase did not follow this same pattern (Fig. 2b). The initial values showed an increase from 57.29%±0.93% (0 Gy) to 68.15%±0.95% (1 Gy) with a significance of *p* < 0.01. Then, the values decreased: 61.97%±1.03% (5 Gy), 51.40%±1.08% (10 Gy) and 40.12%±1.58% (15 Gy), with all differences significant (*p* < 0.01). Compared with 5 Gy, the percentages of cells in S and G2 phases for 10 Gy increased significantly (S, *p* < 0.05*;* G2, *p* < 0.01).

### Cell apoptosis

Flow cytometry was performed to evaluate the apoptotic rates for the glioma cells. Cell apoptosis increased in a radiation dose-dependent manner, as shown in Fig. 2c. Differences in apoptotic rates were observed between C6 and U251 at 0 Gy (*p* < 0.01), 1 Gy (*p* < 0.01), 5 Gy (*p* < 0.05) and 10 Gy (*p* < 0.01). However, when X-ray irradiation increased to 15 Gy, no significant difference was indicated (*p* > 0.05).

### Levels of p-PKC-ι

**Fig. 3:**
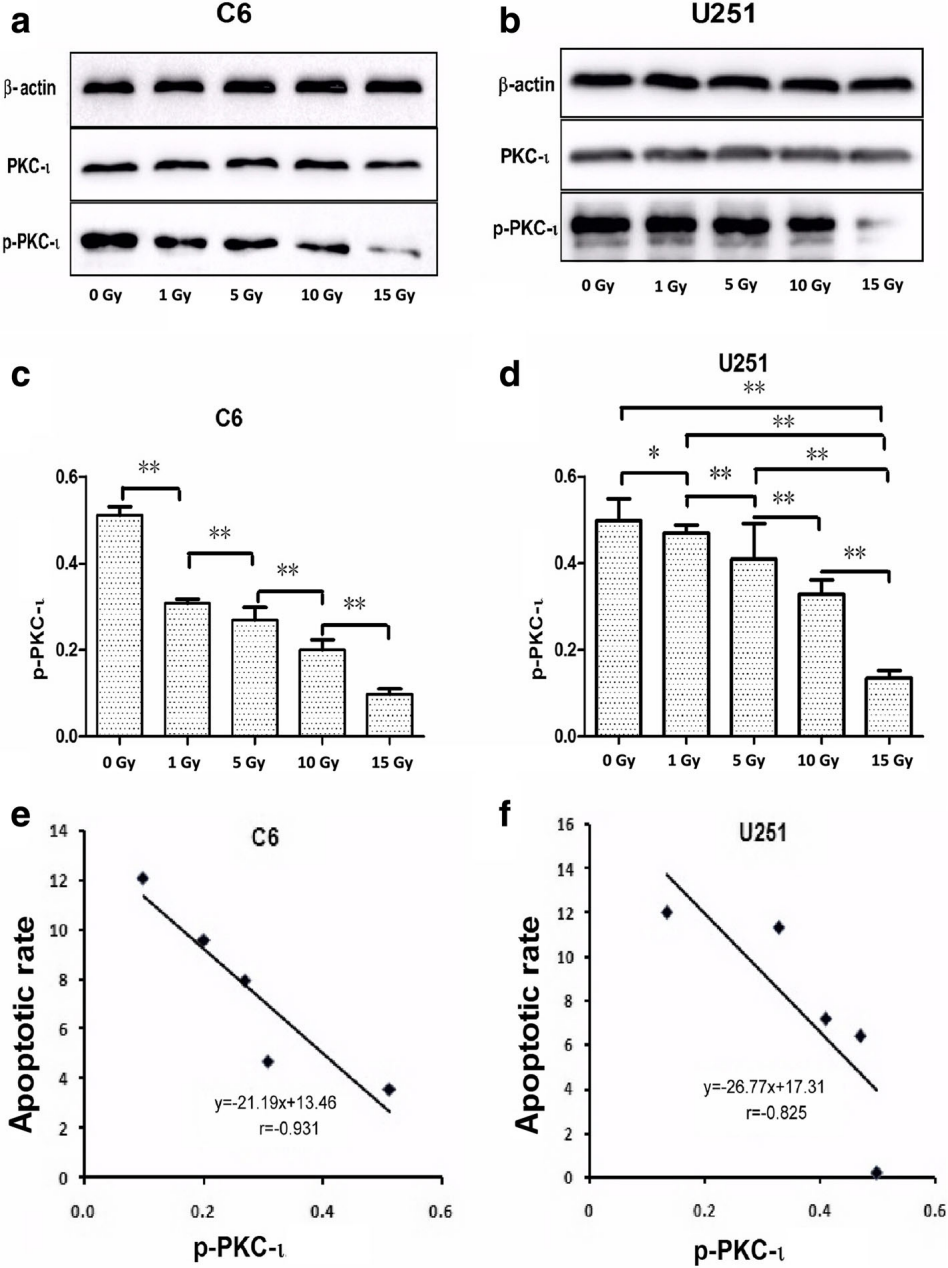
The effects of X-ray irradiation on PKC-ι and p-PKC-ι expression levels. **a** and **b** – Photos of the western blot gels showing the PKC-ι and p-PKC-ι expression levels after C6 and U251 cells were exposed to X-ray irradiation at the indicated doses. **c** and **d** – Results for C6 and U251 showing thep-PKC-ι level normalized to total PKC-ι. The p-PKC-ι expression levels became lower with increasing X-ray irradiation. Statistical data were analyzed using SPSS 17.0. *p < 0.05, **p < 0.01. **e** and **f** – Pearson correlation analysis was performed to determine the correlation between p-PKC-ι and apoptotic rate. A negative linear correlation was found. The linear correlation coefficients were determined as r_C6_ = − 0.931 and r_U251_ = − 0.825

As seen in Fig. 3, the p-PKC-ι level of C6 and U251 cells decreased markedly with increasing X-ray dose (C6, *p* < 0.01; U251, *p* < 0.05). In U251 cells, the differences between 15 Gy and 0, 1, 5, 10 Gy were more discernible (*p* < 0.01). However, the expression of total PKC-ι in C6 and U251 cells only changed slightly.

Pearson correlation also indicated that the p-PKC-ι levels and apoptotic rates exhibited a negative linear correlation, as shown in Fig. 3e and f. The linear correlation coefficients were r_C6_ = − 0.931 and r_U251_ = − 0.825.

### Correlation between p-PKC-ι level and lac/Cr ratio

**Fig. 4:**
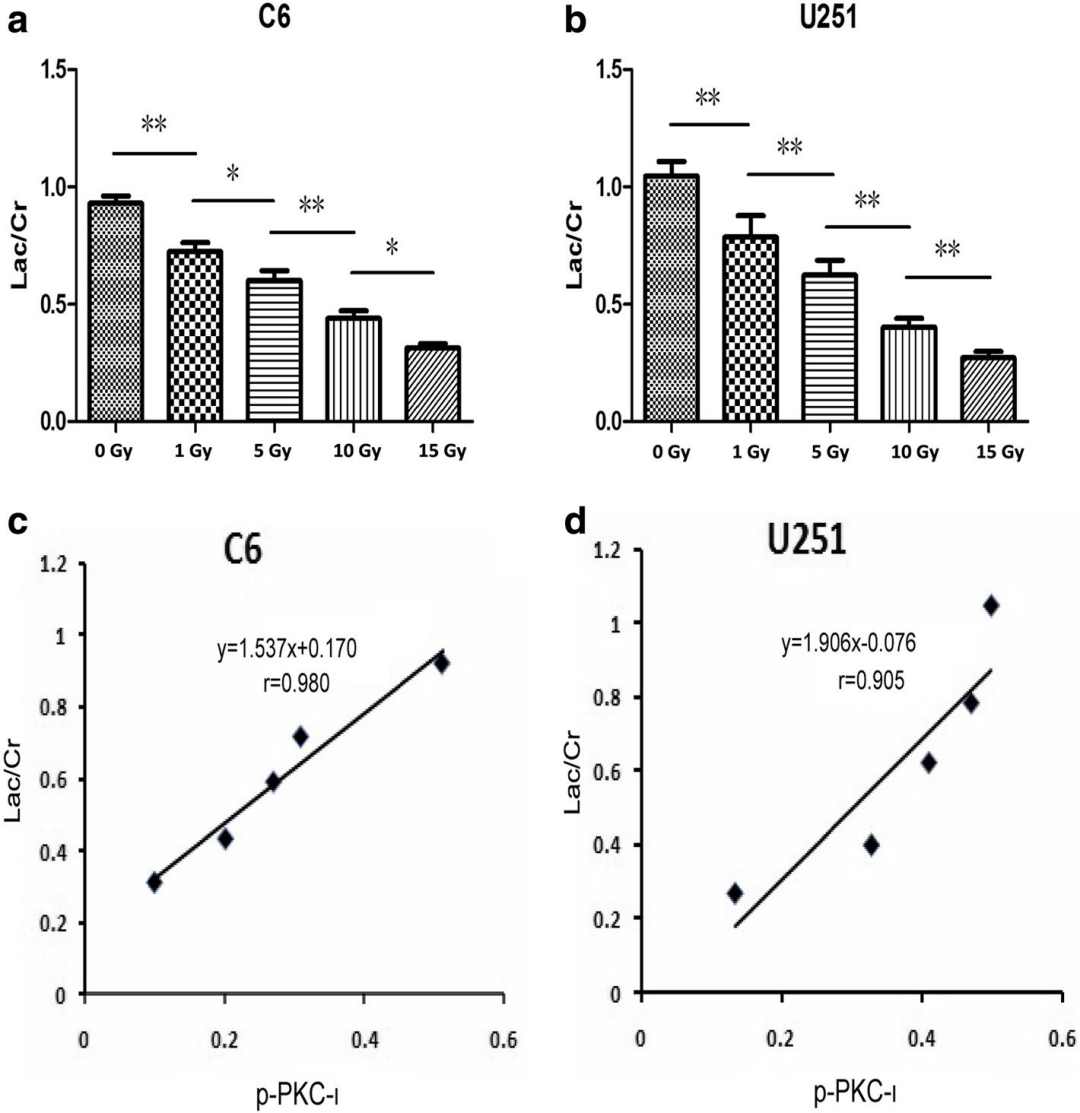
The Lac/Cr ratios were detected using ^1^H NMR spectroscopy and analyzed using MestreNova software. **a** and **b** – With increasing X-ray irradiation, the Lac/Cr ratios of C6 and U251 decreased significantly. **c** and **d** – The Pearson’s correlations between the p-PKC-ι level and Lac/Cr ratio were analyzed using SPSS 17.0. The p-PKC-ι level and the Lac/Cr ratio exhibited a positive linear correlation (r_C6_ = 0.980 and r_U251_ = 0.905)

As shown in Fig. 4a and b, with an increase in X-ray irradiation, the Lac/Cr ratios of C6 and U251 decreased significantly (each *p* < 0.05).The Pearson correlation between the p-PKC-ι level and the Lac/Cr ratio exhibited a positive linear correlation (r_C6_ = 0.980, r_U251_ = 0.905), as analyzed using SPSS 17.0 (Fig. 4c and d).

## Discussion

To further our ability to noninvasively evaluate the effects of radiotherapy, we focused here on confirming that the Lac/Cr ratio correlated with apoptosis in glioma cells and that the ^1^H NMR spectroscopy is a viable means to monitor the Lac/Cr ratio.

Our results confirm that DNA damage and apoptotic rate for glioma cells both increase in an X-ray radiation dose-dependent manner. We also confirmed that the p-PKC-ι level positively correlated with the Lac/Cr ratio and that both negatively correlated with the apoptotic rate. The Lac/Cr ratio was successfully noninvasively monitored via ^1^H NMR spectroscopy.

DNA damage could cause or enhance cell apoptosis [21, 22]. DNA damage leads to cell cycle arrest, which isassociated with apoptosis [12, 13]. As shown in Fig. 2b, the percentage of cells in G1 phase increased significantly in a radiation dose-dependent manner for the C6 cell line. For the U251 cell line, the percentage of cells in G1 phase increased significantly when the X-ray radiation dose increased from 0 Gy to 1 Gy. As shown in Fig. 2c, cell apoptosis in the 2 cell lines increased with increasing X-ray radiation dose. The DNA damage and apoptosis results together show that cell apoptosis increased with increasing DNA damage and that this was associated with G1 phaseblockage. However, the U251 cells displayed some differences:

i.) A decrease in the percentage of cells in G1 phase as irradiation increased to 5 Gy and above
ii.) An increase in the percentage of cells in S phase when comparing the results for 5 and 10 Gy and 10 and 15 Gy
iii.) An increase in the percentage of cells in G2 phase when comparing the results for 5 and 10 Gy and 10 and 15 Gy

These changes indicated that X-ray irradiation-induced DNA damage could lead to cell cycle arrestat different stages (C6: G1 arrest; U251:G1, S or G2 arrest). A previous study similarly revealed that caudatin-induced DNA damage could cause G1, S or G2 arrest in glioma cells [23].

Kikuchi et al. indicated that PKC-ι was over expressed in tumor cells and that it significantly affected tumorigenesis [24]. Furthermore, they showed that chemotherapeutics could reduce the expression of PKC-ι and control apoptosis in tumor cells. Yang et al. similarly suggested that the radiosensitizing effects of tamoxifen on glioma cells were partly attributable to the inhibition of PKC-ι activity in vitro [4]. Here, X-ray irradiation caused a reduction in PKC-ι expression, and this correlated with cell apoptosis. We demonstrated that p-PKC-ι expression decreased markedly in a radiation dose-dependent manner (Fig. 3a–d) and was negatively correlated with cell apoptosis (Fig. 3e and f). With X-ray irradiation at 0 Gy, the p-PKC-ι expression of U251 cells was lower than that of C6 cells, but with X-ray irradiation at 1, 5, 10 and 15 Gy, the p-PKC-ι expressions of U251 cells were higher than those of C6 cells. A previous study demonstrated that drug-mediated PKC-ι inhibition might lead to the activation of pro-apoptotic B-cell lymphoma 2 (associated death promoter) and the dephosphorylation of cyclin-dependent kinase 7, which contributed to increasing cell apoptosis [4]. Results from previous studies [4, 6, 7, 24] and our experiments suggest that PKC-ι inhibition could be partly responsible for the difference in apoptotic rates between the 2 glioma cell lines.

The Wartburg effect shows that cancer is a metabolic disease and that many tumor cells produce energy via glycolysis during cell proliferation [25–27]. Many researchers have attempted to use metabolites to aid in the monitoring of tumor cell treatment in the central nervous system with some success [15, 16]. Glycolysis provides energy for tumor cells and produces lactate [17]. Changes in lactate levels are closely connected with the proliferation and apoptosis rates of glioma cell lines [9, 15]. Creatineis a stable molecule in nerve cells and nervous system tumors, and is employed here as an internal reference.

Our study demonstrated that the lactate-to-creatine (Lac/Cr) ratios of C6 and U251 cells decreased significantly with an increase in X-ray irradiation. Compared with the control group, significant changes occurred in each treatment group of the 2 cell lines. As can be seen by combining the results shown in Fig. 2c with those in Fig. 4a and b, the Lac/Cr ratio negatively connected with the apoptotic rate. This is consistent with the results of previous research. In our control groups, the Lac/Cr ratios of C6 (0.93±0.02) and U251 (1.06 ±0.06) were different. In the 1 Gy and 5 Gy groups, the Lac/Cr ratios of U251 were higher than those of C6. But in the 10 Gy and 15 Gy groups, the Lac/Cr ratios of U251 were lower than those of C6.The heterogeneity of tumors was in part responsible for the differences in the metabolite ratios between C6 and U251 cells. As Martinez-Bisbal et al. demonstrated, extensive heterogeneity can complicate the correlation between tumor biochemical modifications and histopathological features [28]. The relationship between Lac/Cr and p-PKC-ι was also assessed. The results show that the Lac/Cr ratio increased as p-PKC-ι increased, providing further evidence that X-ray irradiation induces changes in metabolites and increases apoptotic rates.

## Conclusions

The lactate-to-creatine (Lac/Cr) ratio decreases with an increase in X-ray irradiation, negatively correlates with the apoptotic rate, and positively correlates with p-PKC-ι expression. This means that the Lac/Cr ratio can be used as a biomarker to reflect the effects of X-ray irradiation in glioma cell lines in culture. Further research is needed to evaluate its potential in a clinical setting.

## Abbreviations

^1^H NMR: Proton nuclear magnetic resonance
Lac/Cr: Lactate-to-creatine
PKC: Protein kinase C
p-PKC-ι: Phosphorylated protein kinase C iota
SCGE: Single cell gel electrophoresis
TMSP: 2,2,3,3-d(4)-3-(trimethylsilyl) propionic acid sodium salt
